# Supplementary material for: Genome-Wide Identification of the BTB Domain-Containing Protein Gene Family in Pepper (Capsicum annuum L.)
Source: Int J Mol Sci. 2025 Apr 6;26(7):3429. doi: 10.3390/ijms26073429 (PMC11989735; doi:10.3390/ijms26073429)
Supplement: Supplementary file 1 [file ijms-26-03429-s001.zip › Supplementary Figures.pdf]

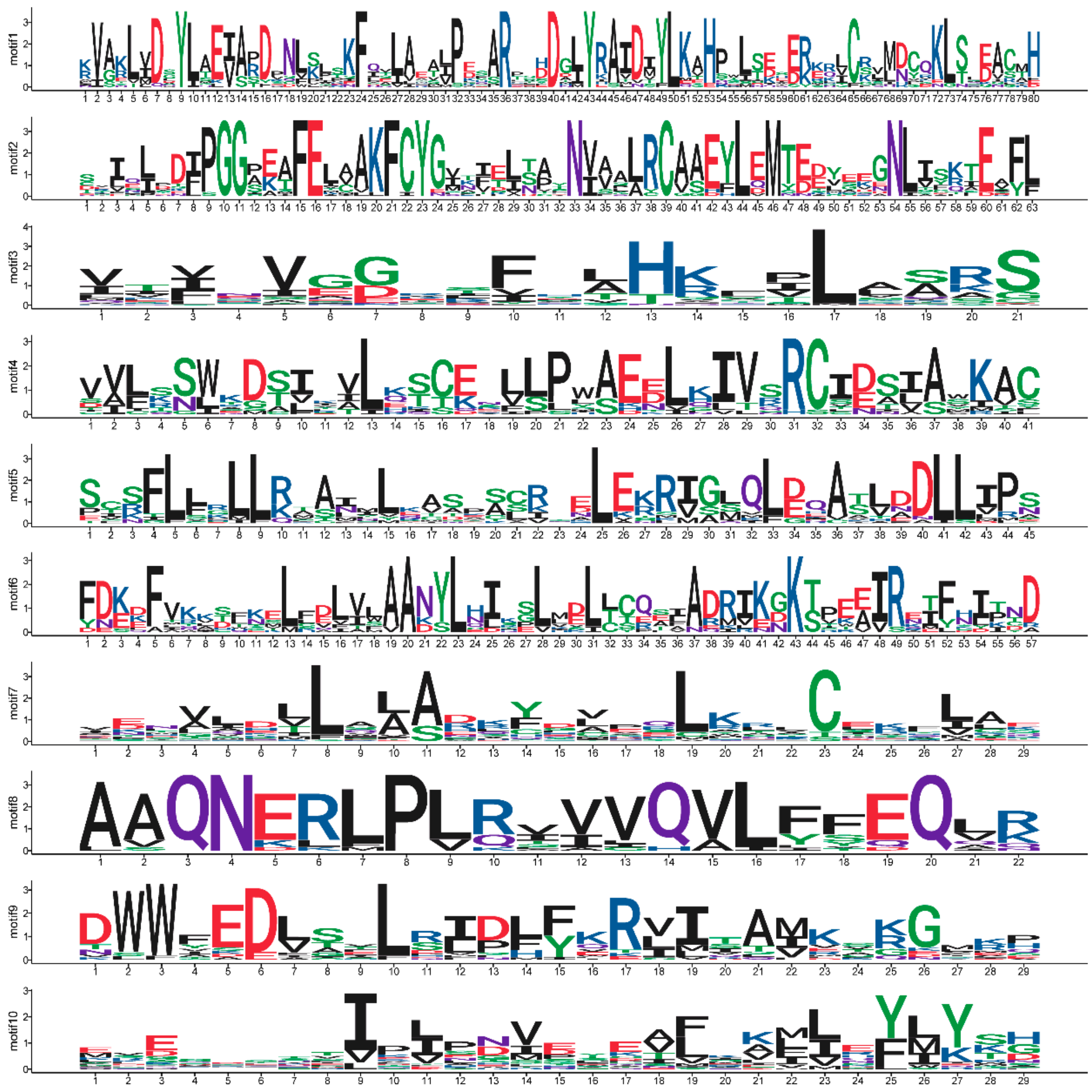

Figure S1. Amino acid sequence logos of the motifs depicted in vibrant letters. Ten representative conserved motifs were discovered in *CaBTB* gene family.

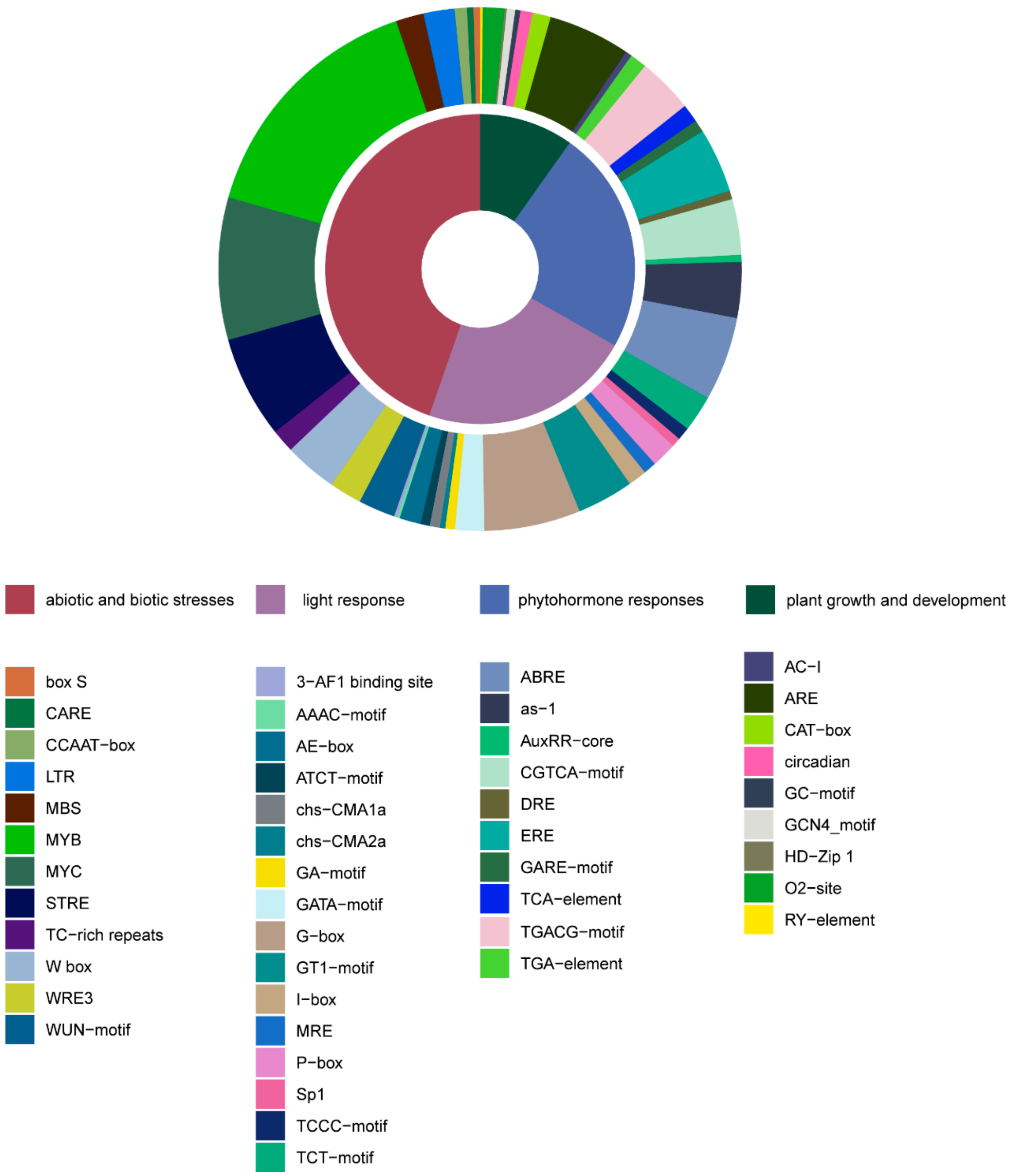

Figure S2. Different types of *cis*-elements identified in the *CaBTB* promoters.
